# Taking insight into the gut microbiota of three spider species: No characteristic symbiont was found corresponding to the special feeding style of spiders

**DOI:** 10.1002/ece3.5382

**Published:** 2019-06-23

**Authors:** Guowen Hu, Lihua Zhang, Yueli Yun, Yu Peng

**Affiliations:** ^1^ State Key Laboratory of Biocatalysis and Enzyme Engineering, Hubei Collaborative Innovation Center for Green Transformation of Bio‐Resources, School of Life Sciences Hubei University Wuhan China; ^2^ Centre for Behavioral Ecology & Evolution, School of Life Sciences Hubei University Wuhan China

**Keywords:** *Burkholderia*, gut microbiota, *Ralstonia*, *Rickettsiella*, spider, *Wolbachia*

## Abstract

Microorganisms in insect guts have been recognized as having a great impact on their hosts' nutrition, health, and behavior. Spiders are important natural enemies of pests, and the composition of the gut microbiota of spiders remains unclear. Will the bacterial taxa in spiders be same as the bacterial taxa in insects, and what are the potential functions of the gut bacteria in spiders? To gain insight into the composition of the gut bacteria in spiders and their potential function, we collected three spider species, *Pardosa laura*, *Pardosa astrigera,* and *Nurscia albofasciata*, in the field, and high‐throughput sequencing of the 16S rRNA V3 and V4 regions was used to investigate the diversity of gut microbiota across the three spider species. A total of 23 phyla and 150 families were identified in these three spider species. The dominant bacterial phylum across all samples was Proteobacteria. *Burkholderia*, *Ralstonia*, *Ochrobactrum*, *Providencia*, *Acinetobacter*, *Proteus,* and *Rhodoplanes* were the dominant genera in the guts of the three spider species. The relative abundances of *Wolbachia* and *Rickettsiella* detected in *N*. *albofasciata* were significantly higher than those in the other two spider species. The relative abundance of *Thermus*, *Amycolatopsis*, *Lactococcus*, *Acinetobacter Microbacterium,* and *Koribacter* detected in spider gut was different among the three spider species. Biomolecular interaction networks indicated that the microbiota in the guts had complex interactions. The results of this study also suggested that at the genus level, some of the gut bacteria taxa in the three spider species were the same as the bacteria in insect guts.

## 1 INTRODUCTION

Gut bacteria are a vital bacterial community in arthropods and have been reported in regard to their compositions and potential functions (Engel, Martinson, & Moran, 2012; Hongoh, 2011; Thong‐On et al., 2012). In recent years, researchers have become increasingly interested in the gut bacteria of arthropods with the advent of next‐generation sequencing (NGS) technology. The diversity of gut bacteria has been studied in a range of insects and ticks (Anjum et al., 2018; Muturi, Ramirez, Rooney, & Kim, 2017; Snyman, Gupta, Bezuidenhout, Claassens, & Van dBJ, 2016; Waite et al., 2015). Gut bacteria have a great impact on their insect hosts, such as resistance against parasites and pathogens (Dillon, Vennard, & Charnley, 2005; Koch & Schmid‐Hempel, 2011), nutrient supplementation (Eichler & Schaub, 2002; Hongoh et al., 2008), intestinal cell renewal and developmental rate (Shin et al., 2011), and digestion of dietary compounds (Gaio et al., 2011). In addition, gut bacteria can produce molecules for communication within and between species (Leroy et al., 2011; Sharon et al., 2010). Thus, the gut microbiota could be considered a bacterial organ that is integrated into the biological system of the host (Bäckhed, Ley, Sonnenburg, Peterson, & Gordon, 2005).

However, little is known about the gut bacterial communities of spiders, which are viewed as one of the major groups of generalist predators in sustainable agricultural systems (Ekschmitt, Wolters, & Weber, 1997). Spiders belong to the phylum Arthropoda, class Arachnida, and order Araneae, and they are widely distributed throughout the world. Some spider species are important natural enemies of agricultural pests, which play crucial roles in pest biological control in paddy fields, orchards, cotton fields, and tea gardens (Marc, Canard, & Ysnel, 1999; Michalko & Pekár, 2015; Nyffeler & Sunderland, 2003). Most of the studies on microorganisms within spiders have focused on endosymbionts and their reproductive effects on their hosts (Duron, Hurst, Hornett, Josling, & Engelstädter, 2008; Goodacre, Martin, Thomas, & Hewitt, 2006; Rowley, Raven, & McGraw, 2004). Endosymbionts (such as *Wolbachia*, *Cardinium*, *Spiroplasma,* and *Rickettsia*) prevail in some spider hosts, and some of them have a great effect on spider reproduction by inducing sex ratio variations (Gunnarsson, Goodacre, & Hewitt, 2009; Vanthournout & Hendrickx, 2015; Vanthournout, Swaegers, & Hendrickx, 2011; Vanthournout, Vandomme, & Hendrickx, 2014; Zhang, Yun, Hu, & Peng, 2018). Although much research has been conducted on endosymbionts in spiders, gut bacteria, as a very important part of bacterial communities in spiders, have rarely been reported on. Moreover, spiders have their own special feeding style compared with insects and other arthropods. They usually bite part of the prey and then quickly inject venom into the body of prey and feed suctorially (Foelix, 2011). The following is the process of the spider's predation: (a) locating the prey; (b) turning toward the victim and grasping it with the tips of the front legs; (c) pulling the prey to the chelicerae and biting it (venom injection); (d) releasing the grasp with the legs and holding the prey only with the chelicerae; (e) fastening some silk threads over the immobilized prey; and (f) feeding (Foelix, 2011). Therefore, according to the special predatory way of spiders, we hypothesize that the gut bacterial communities of spiders may be different from those of other arthropods because of their special feeding style.

In an effort to explore the gut bacterial communities of spiders, we selected three species, *Pardosa laura* (Lycosidae), *Pardosa astrigera* (Lycosidae), and *Nurscia albofasciata* (Titanoecidae), as focal species in this study. *P. laura*, *P. astrigera,* and *N. albofasciata* are common spiders in cotton fields (Zhao, 1993); thus, they have similar habitats and are easy to collect. *P. laura* and *P. astrigera* are wandering predators that do not build webs, while *N. albofasciata* is a species of web‐building spider; thus, the spider species from the two genera have different methods of predation.

Identifying the diversity of the spider gut microbiota is the first step in understanding the relationship between the spiders and their gut microbiota. Therefore, the aim of this study was to characterize the gut microbiota associated with the spider species (*P*.* laura*, *P*. *astrigera,* and *N*. *albofasciata*) by Illumina sequencing the 16S rRNA V3‐V4 high variable region and to compare the gut bacterial components of different spider species. Statistical analyses were performed to identify microbial interactions and to elucidate the potential function of those microbes. This study is an early attempt to examine the gut microbiota of spiders, and it will provide a foundation for future studies on the relationships between gut microbiota and their hosts.

## 2 MATERIALS AND METHODS

### 2.1 Samples

In this study, *N*. *albofasciata*, *P*. *astrigera,* and *P*. *laura* were collected in the cotton field of Huazhong Agricultural University, Wuhan, China. All samples were collected during the same season. All living samples were transported to the laboratory and starved for at least 7 days before dissection to remove the non‐native microorganisms in the spider guts. The samples were visually identified under microscopes. Before dissection, the samples were first rinsed in sterile water, surface sterilized with 70% ethanol for 5 min, and then washed three times with sterile water. The hindgut was dissected from each individual in sterile phosphate‐buffered saline (PBS) solution with sterile forceps under a microscope, placed into 1.5‐ml microcentrifuge tubes, washed three times with sterile water, and finally stored in a −80°C freezer. The spiders used in this study were all identified as nonendangered and nonprotected species.

### 2.2 DNA extraction and 16S rRNA gene amplicon sequencing

We pooled six guts into each sample. Three biological replicates were set for each sample. The total DNA of each pooled sample was extracted using a DNeasy Blood & Tissue Kit (Qiagen) following the manufacturer's protocol. Each DNA sample was amplified using the universal 16S rRNA gene primers (27F: 5'‐AGAGTTTGATCATGGCTCAG‐3' and 1487R: 5'‐TACCTTGTTACGACTTCACC‐3'; Heddi, Grenier, Khatchadourian, Charles, & Nardon, 1999). PCR amplification was carried out in a total volume of 30 μl containing 1 μl of each primer, 0.5 μl of template DNA, 0.5 μl of Taq DNA polymerase, 1 μl of dNTPs, 3 μl of 10 × buffer, and 23 μl of sterile distilled water. The following parameters were used in the PCRs: denaturation for 5 min at 94°C and 35 cycles of denaturation for 30 s at 94°C, annealing for 45 s at 53°C, and elongation at 72°C for 45 s. For the last cycle, the elongation time was extended to 7 min at 72°C. PCR products were run on 2% agarose gels, and samples producing visualized amplicons were utilized for high‐throughput sequencing of microbial diversity. The variable V3–V4 region of the 16S rDNA was used to assess bacterial diversity. Sequencing was carried out on an Illumina MiSeq platform at Personal Biotechnology Co., Ltd.

### 2.3 Bioinformatic processing and statistical analyses

Paired‐end reads were merged into single reads by FLASH version 1.2.7 (Magoc & Salzberg, 2011). High‐quality clean tags were obtained by Trimmomatic version 0.33 through quality filtering on the Raw tags (Bolger, Lohse, & Usadel, 2014). UCHIME version 4.2 (default setting: 80% similarity) was used to identify and remove chimeric sequences (Edgar, Haas, Clemente, Quince, & Knight, 2011). The remaining sequences were clustered into operational taxonomic units (OTUs) at 97% similarity using UCLUST version 1.2.22 (Edgar, 2010). Taxonomic assignment of each OTU was carried out by aligning representative sequences (the sequences that have the highest relative abundances) of each cluster to references from Greengenes (Release 13.8; DeSantis et al., 2006). The raw reads were submitted to the NCBI Sequence Read Archive (SRA) database (Accession number: SRP149550).

All statistical analyses were conducted using the R statistical computing environment (R version 3.3.1; R Development Core Team, 2016). Alpha‐diversity analysis metrics including bacterial richness (observed OTUs and Chao 1 estimators) and bacterial diversity (Shannon and Simpson index) were calculated with Mothur software (version V.1.3.0). The Mann–Whitney *U* test was used to test for difference in these index values between two different groups. Differences in relative abundances of certain bacterial species were analyzed among different groups using one‐way ANOVA in SPSS BASE version 19.1 statistical software (SPSS; Ahn et al., 2012). The differences were considered significant when *p* < 0.05. Principal component analysis (PCA) was conducted to explore the differences in bacterial communities at the genus level across different samples.

### 2.4 Ecology network analyses and functional predictions

To investigate how the gut bacteria interacted with each other, a correlation matrix of bacteria was constructed by Spearman's rank correlations. The Biomolecular Interaction Networks were used to predict the interactions of gut bacteria (positive or negative; Shannon et al., 2003). The networks were constructed using RMT models after taking logarithmic and Pearson correlation estimation (Zhou, Deng, Luo, He, & Yang, 2011). We used Mothur software to calculate Spearman's rank correlation coefficients for the top 50 genera of bacteria. The qualified genera (*ρ* > 0.6 and *p* < 0.01) were retained for network construction, and the network was visualized with Cytoscape software (Shannon et al., 2003).

Phylogenetic Investigation of Communities by Reconstruction of Unobserved States (PICRUSt) is a bioinformatic tool that uses the 16S rRNA gene to predict the abundance of functional genes by matching sample OTUs with reference genomes (Greengenes; Langille et al., 2013). Then, the prediction for microbial metabolism was categorized into the Kyoto Encyclopedia of Genes and Genomes (KEGG) at level 2 (Kanehisa, Goto, Sato, Furumichi, & Tanabe, 2012). In addition, the prediction accuracy of PICRUSt was evaluated by the Nearest Sequenced Taxon Index (NSTI), with a low value indicating a high prediction accuracy (Langille et al., 2013).

## 3 RESULTS

### 3.1 The diversity analysis of the bacterial community

**Figure 1:**
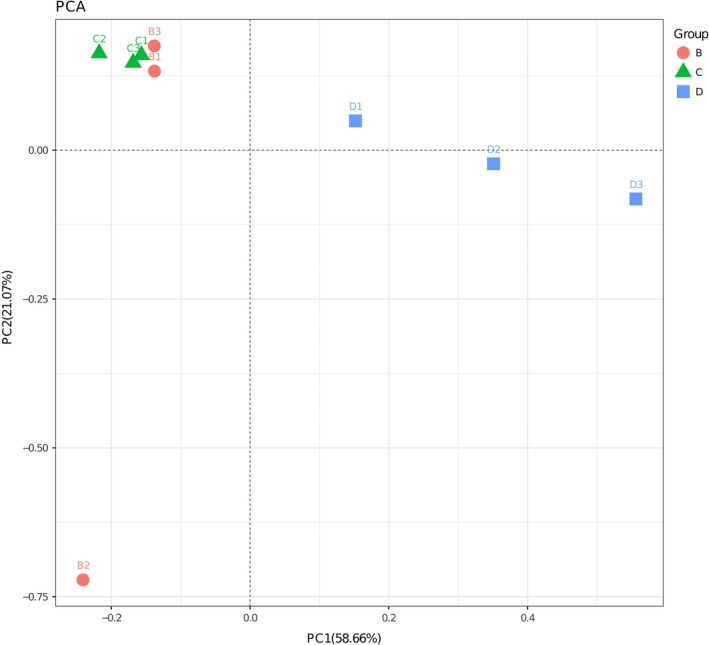
PCA for visualization of gut bacterial community dissimilarities. Each point represents a sample, and points of different colors belong to different samples. The distance between two points represents their level of similarity (B = *P. laura*, C = *P. astrigera*, and D = *N. albofasciata*)

The Illumina MiSeq sequencing of the 16S rRNA gene amplicons from the field samples of the spiders yielded 312,800 raw reads per sample. After quality filtering and the removal of chimeric sequences, 298,402 sequences were retained, with a mean of 33,156 reads per sample remaining. The three spider species had a high number of OTUs ranging from 861.00 ± 76.49 to 1,177.67 ± 263.85. The results of the statistical analyses for richness and diversity indices showed that no significant differences were detected between any two spider hosts (*p* > 0.05, see Table 1). The PCA plot showed that the bacterial communities were much more similar within species than between species (Figure 1).

**Table 1:** Significant test (*p* values) of alpha diversity and OTUs between three samples

Taxon	B‐C	B‐D	C‐D
Shannon	0.689	0.140	0.247
Simpson	0.683	0.208	0.363
Chao 1	0.950	0.333	0.361
OTUs	0.847	0.240	0.31

Note

*p* < 0.05 indicates significantly difference. (B = *P*. *laura*, C = *P*. *astrigera*, and D = *N*. *albofasciata*.)

### 3.2 Composition of bacterial community

**Figure 2:**
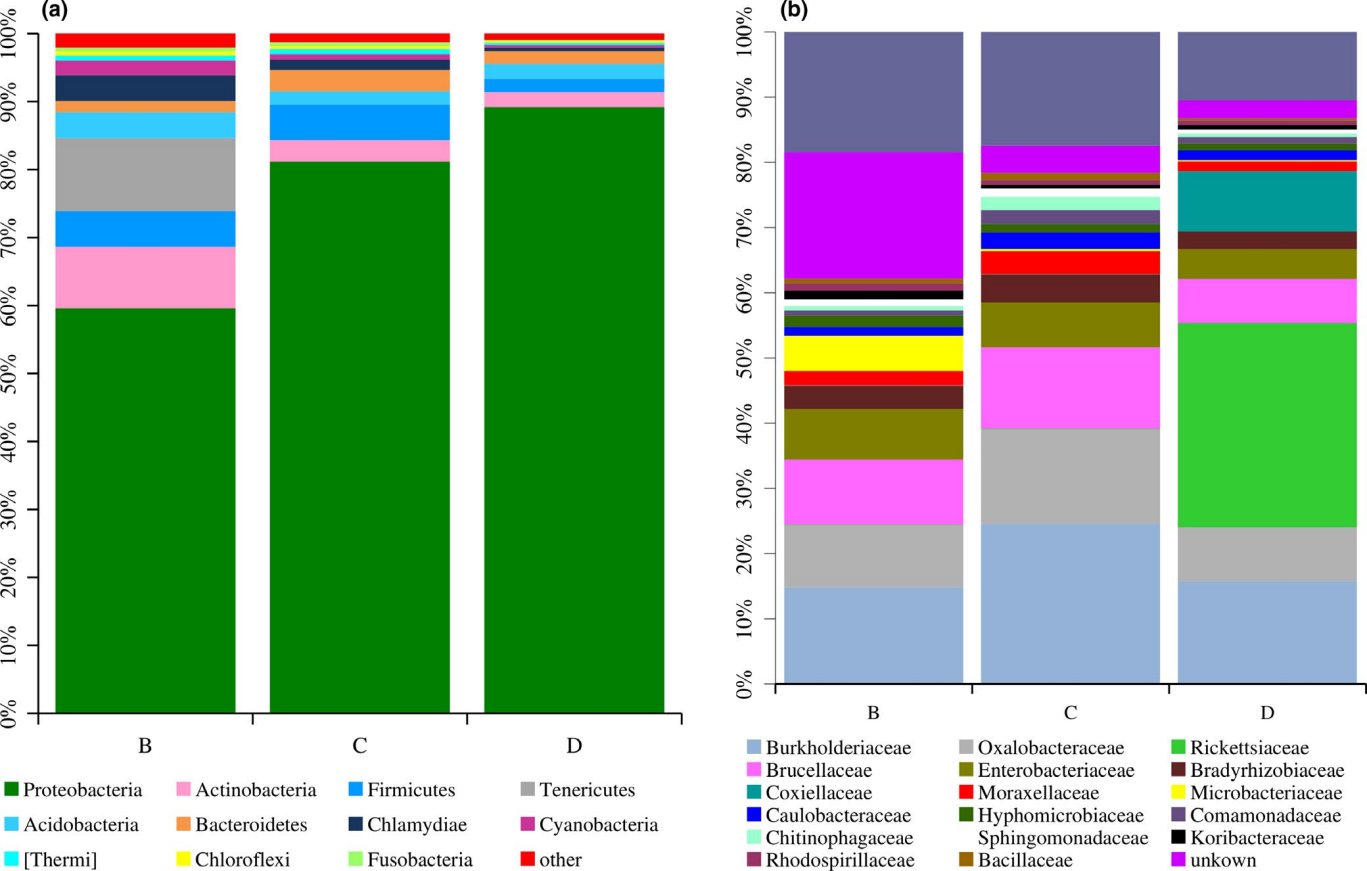
Relative abundance of dominant bacterial taxa at the phylum level and at the family level (B = *P. laura*, C = *P. astrigera*, and D = *N. albofasciata*)

All high‐quality reads were clustered into 23 phyla and 150 families in the present spider species. The most dominant phylum was Proteobacteria (Figure 2a), which varied significantly between the different spider species (*p* < 0.05; see Table 2). The relative abundance of Proteobacteria accounted for 59.59%, 81.16%, and 89.19% of the total reads in *P*. *laura*, *P*. *astrigera,* and *N*. *albofasciata*, respectively. In addition, other bacterial taxa, including Tenericutes, Actinobacteria, Firmicutes, Acidobacteria, and Bacteroidetes, were also detected in the guts of the three spider species (see Figure 2a). At the family level, Burkholderiaceae, Oxalobacteraceae, Brucellaceae, Enterobacteriaceae, Bradyrhizobiaceae, Moraxellaceae, Caulobacteraceae, and Hyphomicrobiaceae were the core bacterial taxa within three spider species (Figure 2b). Rickettsiaceae (31.33%) and Coxiellaceae (9.25%) had a significantly higher relative abundance in *N*. *albofasciata* compared to those of *P*. *laura* and *P*. *astrigera* (*p* = 0.002, *p < *0.001; see Table 3). At the genus level, 237 genera were detected in the three spider species. Some bacterial genera with an abundance ≥ 1% were detected, and *Burkholderia*, *Ralstonia*, *Ochrobactrum*, *Rickettsiella, Providencia*, *Acinetobacter*, *Proteus,* and *Rhodoplanes* comprised the major gut bacterial taxa. *Wolbachia* (30.13%) and *Rickettsiella* (9.24%) had a significantly higher relative abundance in *N*. *albofasciata* compared to those of *P*. *laura* and *P*. *astrigera* (*p* < 0.05; Table 4).

**Table 2:** Significant test (*p* values) of selected phylum which total abundance >1% between three samples

Phylum	B‐C	B‐D	C‐D
Proteobacteria	0.004	0.001	0.149
Actinobacteria	0.211	0.153	0.824
Firmicutes	0.994	0.050	0.051
Tenericutes	0.266	0.266	0.988
Acidobacteria	0.276	0.340	0.876
Bacteroidetes	0.186	0.822	0.255
Chlamydiae	0.349	0.191	0.663
Cyanobacteria	0.296	0.188	0.744

Note

*p* < 0.05 indicates significantly difference. (B = *P*. *laura*, C = *P*. *astrigera*, and D = *N*. *albofasciata*.)

**Table 3:** Significant test (*p* values) of selected family which total abundance in the top 30 between three samples

Family	B‐C	B‐D	C‐D
Burkholderiaceae	0.136	0.876	0.170
Oxalobacteraceae	0.138	0.688	0.077
Rickettsiaceae	0.998	0.002	0.002
Brucellaceae	0.376	0.269	0.073
Enterobacteriaceae	0.580	0.080	0.178
Bradyrhizobiaceae	0.266	0.184	0.034
Coxiellaceae	0.994	0.000	0.000
Moraxellaceae	0.131	0.370	0.035
Microbacteriaceae	0.276	0.264	0.975
Caulobacteraceae	0.126	0.869	0.160
Hyphomicrobiaceae	0.428	0.263	0.713
Comamonadaceae	0.083	0.795	0.129
Chitinophagaceae	0.060	0.850	0.046
Sphingomonadaceae	0.558	0.358	0.157
Koribacteraceae	0.202	0.294	0.785
Rhodospirillaceae	0.386	0.345	0.932
Bacillaceae	0.363	0.371	0.099
Thermaceae	0.895	0.032	0.039
Lactobacillaceae	0.940	0.153	0.170
Pseudomonadaceae	0.103	0.926	0.090
Ruminococcaceae	0.131	0.027	0.288
Fusobacteriaceae	0.959	0.229	0.214
Methylobacteriaceae	0.685	0.419	0.244
Planococcaceae	0.402	0.676	0.229
Pseudonocardiaceae	0.357	0.051	0.201
Alcaligenaceae	0.337	0.597	0.160
Sinobacteraceae	0.902	0.707	0.799
Streptococcaceae	0.106	0.003	0.025
Amoebophilaceae	1.000	0.269	0.269
Propionibacteriaceae	0.794	0.207	0.142

Note

*p* < 0.05 indicates significantly difference. (B = *P*. *laura*, C = *P*. *astrigera*, and D = *N*. *albofasciata*.)

**Table 4:** Comparisons of relative abundance (>0.1%) for gut bacteria across the three spider hosts

Phylum	Genus	B	C	D
Proteobacteria	*Burkholderia*	14.85 ± 2.05	24.56 ± 5.18	15.76 ± 4.08
	*Ralstonia*	9.28 ± 1.33	14.22 ± 3.15	8.15 ± 1.17
	*Wolbachia*	0.09 ± 0.04^a^	0.08 ± 0.02^a^	30.13 ± 6.84^b^
	*Ochrobactrum*	9.97 ± 1.82	12.51 ± 2.60	6.75 ± 0.68
	*Rickettsiella*	0.02 ± 0.01^a^	0.02 ± 0.01^a^	9.24 ± 0.74^b^
	*Providencia*	3.14 ± 0.53	2.26 ± 0.40	1.85 ± 0.34
	*Acinetobacter*	2.15 ± 0.05^ab^	3.36 ± 0.80^a^	1.43 ± 0.33^b^
	*Proteus*	2.30 ± 0.55	1.83 ± 0.14	1.38 ± 0.20
	*Rhodoplanes*	1.54 ± 0.57	1.02 ± 0.03	0.95 ± 0.33
	*Delftia*	0.41 ± 0.04	1.69 ± 0.83	0.84 ± 0.29
	*Rickettsia*	0.01 ± 0.01	0.00 ± 0.00	1.21 ± 0.61
	*Sphingomonas*	0.79 ± 0.31	0.95 ± 0.27	0.48 ± 0.10
	*Pseudomonas*	0.31 ± 0.01	0.56 ± 0.13	0.29 ± 0.06
	*Methylobacterium*	0.41 ± 0.17	0.52 ± 0.28	0.17 ± 0.03
	*Serratia*	0.34 ± 0.05	0.51 ± 0.12	0.25 ± 0.07
	*Achromobacter*	0.23 ± 0.04	0.31 ± 0.01	0.22 ± 0.04
	*Edwardsiella*	0.18 ± 0.01	0.35 ± 0.15	0.13 ± 0.09
	*Aminobacter*	0.17 ± 0.04	0.22 ± 0.06	0.11 ± 0.01
	*Agrobacterium*	0.14 ± 0.04	0.23 ± 0.04	0.13 ± 0.02
	*Luteimonas*	0.08 ± 0.02	0.16 ± 0.09	0.22 ± 0.17
	*Paracoccus*	0.06 ± 0.00	0.21 ± 0.09	0.08 ± 0.04
	*Cellvibrio*	0.08 ± 0.02	0.10 ± 0.03	0.12 ± 0.09
	*Janthinobacterium*	0.10 ± 0.03	0.15 ± 0.05	0.05 ± 0.02
	*Asticcacaulis*	0.09 ± 0.01	0.12 ± 0.07	0.07 ± 0.04
	*Morganella*	0.07 ± 0.02	0.10 ± 0.04	0.04 ± 0.02
	*Bdellovibrio*	0.02 ± 0.00	0.11 ± 0.04	0.07 ± 0.03
	*Lysobacter*	0.01 ± 0.01	0.12 ± 0.09	0.05 ± 0.02
Actinobacteria	*Amycolatopsis*	0.34 ± 0.08^a^	0.27 ± 0.03^ab^	0.16 ± 0.02^b^
	*Propionibacterium*	0.29 ± 0.03	0.32 ± 0.15	0.11 ± 0.03
	*Microbacterium*	0.09 ± 0.01^a^	0.16 ± 0.01^b^	0.09 ± 0.02^a^
Bacteroidetes	*Sediminibacterium*	0.62 ± 0.05	2.03 ± 0.75	0.49 ± 0.04
	*Cardinium*	0.00 ± 0.00	0.00 ± 0.00	0.74 ± 0.74
	*Chryseobacterium*	0.16 ± 0.11	0.10 ± 0.02	0.05 ± 0.02
	*Bacteroides*	0.10 ± 0.04	0.12 ± 0.06	0.04 ± 0.02
Acidobacteria	*Candidatus*	0.34 ± 0.15	0.16 ± 0.02	0.21 ± 0.10
	*Koribacter*	0.21 ± 0.09^a^	0.04 ± 0.01^b^	0.06 ± 0.02^b^
	*Corynebacterium*	0.13 ± 0.02	0.12 ± 0.06	0.04 ± 0.00
	*Brevibacterium*	0.08 ± 0.02	0.10 ± 0.03	0.03 ± 0.01
[Thermi]	*Thermus*	0.71 ± 0.15^a^	0.71 ± 0.09^a^	0.31 ± 0.06^b^
Firmicutes	*Bacillus*	0.46 ± 0.15	0.57 ± 0.12	0.20 ± 0.06
	*Lactobacillus*	0.51 ± 0.18	0.44 ± 0.09	0.25 ± 0.05
	*Cetobacterium*	0.29 ± 0.02	0.47 ± 0.20	0.14 ± 0.06
	*Lactococcus*	0.33 ± 0.04^a^	0.21 ± 0.06^ab^	0.11 ± 0.01^b^
	*Clostridium*	0.22 ± 0.03	0.22 ± 0.07	0.07 ± 0.02
	*Kurthia*	0.21 ± 0.04	0.08 ± 0.01	0.05 ± 0.01
Fusobacteria	*Fusobacterium*	0.26 ± 0.23	0.09 ± 0.04	0.03 ± 0.01

Note

Data are shown as Mean ± *SE*; B = *P. laura*, C = *P. astrigera*, and D = *N. albofasciata*; values with different letters indicate a significant difference (*p* < 0.05).

As shown in Table 4, at the genus level, in addition to the two endosymbionts *Wolbachia* and *Rickettsiella*, there were significant differences in the relative abundance of *Thermus*, *Amycolatopsis*, *Lactococcus*, *Acinetobacter*, *Microbacterium,* and *Koribacter* from the gut bacterial communities of three spider species. The relative abundance of *Wolbachia* and *Rickettsiella* in *N. albofasciata* was significantly higher than those in the other two spider species (*p* < 0.05), while the relative abundance of *Thermus* in *N. albofasciata* was lower than that in the other two species (*p* < 0.05). The relative abundances of *Amycolatopsis* and *Lactococcus* in *P. laura* were significantly higher than that in *N. albofasciata* (*p* < 0.05). *P*. *astrigera* had a higher abundance of *Microbacterium* than the other two species (Table 4). The relative abundance of *Acinetobacter* in *P*. *astrigera* was significantly higher than that in *N. albofasciata* (*p* < 0.05), and the relative abundance of *Koribacter* in *P. laura* was higher than that in other spider hosts (Table 4).

### 3.3 Biomolecular interaction networks and functional predictions with PICRUSt across the gut microbiota of the three spiders

**Figure 3:**
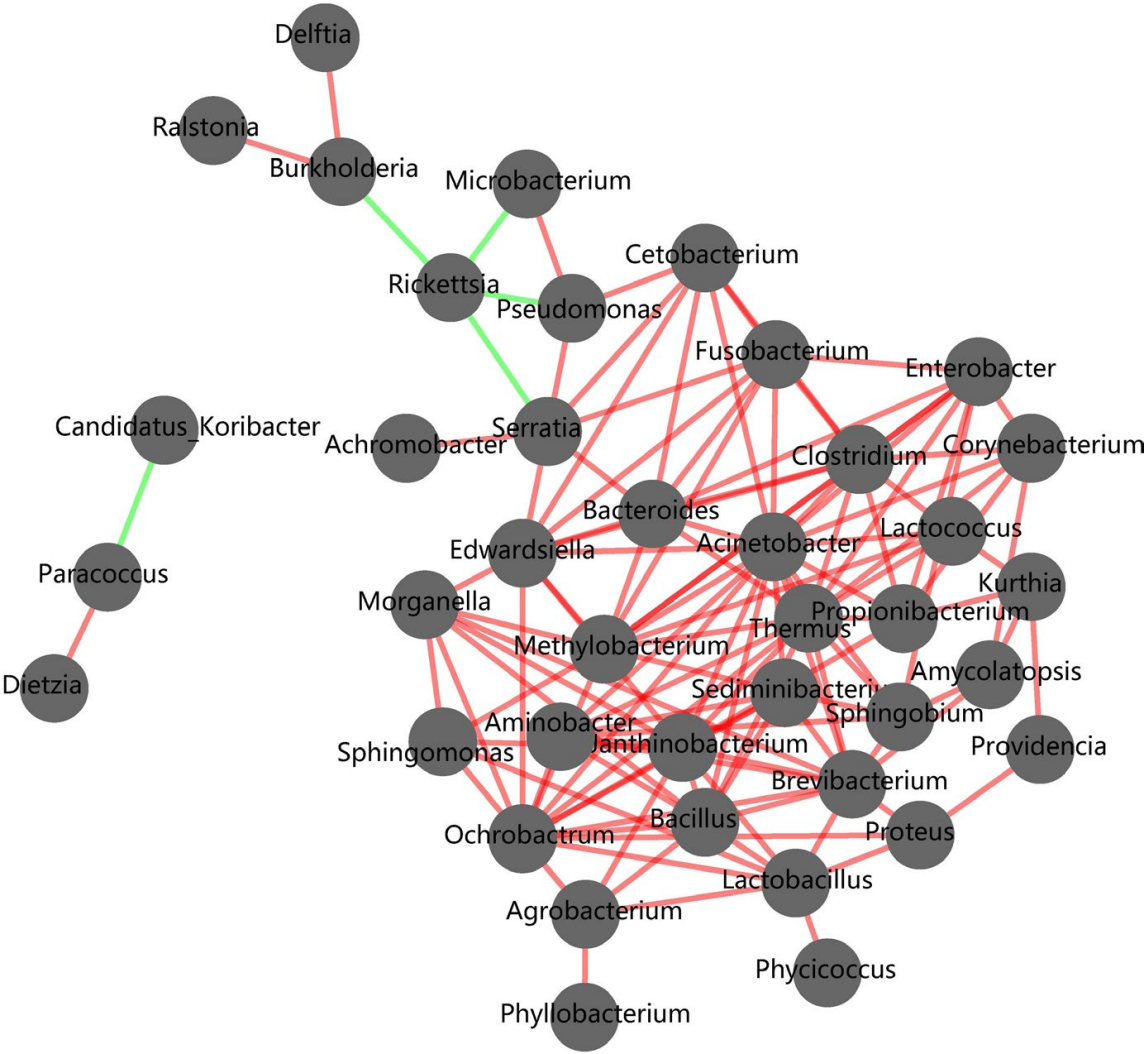
The Biomolecular Interaction Networks of all samples. Nodes represent OTUs, and lines connecting nodes represent positive (light red) and negative (light blue) interactions

**Figure 4:**
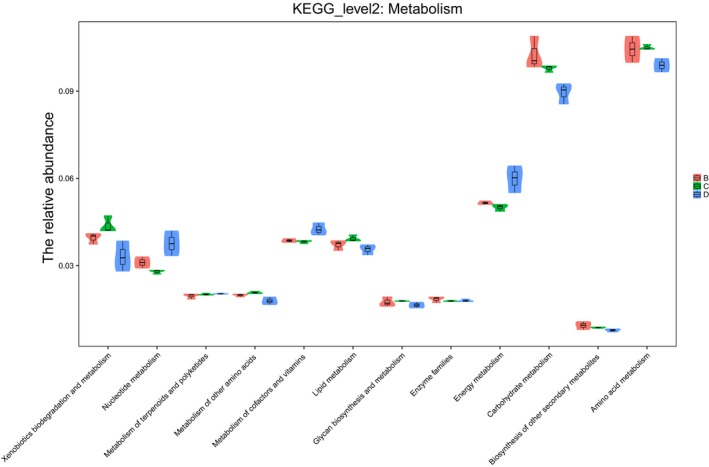
Predicted function of gut microbiota in the three spider species. All KEGG metabolic pathways are shown at the second hierarchical level and grouped by major functional categories. (B = *P. laura*, C = *P. astrigera*, and D = *N. albofasciata*)

The correlations among most of the microbes detected in this study were positive in the biomolecular interaction networks, and only a few negative connections were discovered, such as the correlations between *Rickettsia* and three other bacteria (*Burkholderia*, *Microbacterium,* and *Pseudomonas*) and the correlations between *Candidatus*, *Koribacter,* and *Paracoccus* (Figure 3). PICRUSt provided insights into the potential metabolic functions of the spider gut microbiota. The metabolism pathways of gut bacteria included amino acid metabolism, carbohydrate metabolism, energy metabolism, lipid metabolism, metabolism of cofactors and vitamins, xenobiotics biodegradation and metabolism, nucleotide metabolism, and so on (Figure 4), and the gut bacteria involved in amino acid metabolism and carbohydrate metabolism showed higher relative abundances than the bacteria involved in the other metabolism pathways (Figure 4).

## 4 DISCUSSION

This study compared the composition of gut bacteria across three spider species. A total of 23 phyla and 150 families were identified in the spider hosts, and Proteobacteria was the most dominant bacterial phylum. The bacteria from Proteobacteria are reported to construct the main gut bacterial structures within a variety of insects, such as butterflies (*Spodoptera littoralis*; Chen et al., 2016), moths (*Melitaea cinxia*; Ruokolainen, Ikonen, Makkonen, & Hanski, 2016), bugs (*Manduca sexta*; Hammer, Janzen, Hallwachs, Jaffe, & Fierer, 2017), and bees (Engel et al., 2012; Engel & Moran, 2013). *Burkholderia* and *Ralstonia* were the most dominant bacterial genera detected in the spider guts in this study. *Ralstonia* was also found in bees (Anjum et al., 2018;), and *Burkholderia* was distributed in mosquitoes (Charan et al., 2016), stinkbugs (Kikuchi, Hosokawa, & Fukatsu, 2007), and flies (Vivero, Jaramillo, Cadavid‐Restrepo, Soto, & Herrera, 2016). *Burkholderia* often occurs in the natural environment (Compant, Nowak, Coenye, Clément, & Ait Barka, 2008). Many studies have suggested that *Ralstonia* is a pathogen in plants (Denny, 2007; Morel et al., 2018; Prior et al., 2016). In populations of the stinkbug *Riptortus pedestris*, *Burkholderia* acquired from the environment gained the ability to hydrolyze fenitrothion to protect their hosts (Kikuchi et al., 2012). Some *Burkholderia* strains exhibited nitrogen‐fixing abilities (Estrada‐De, Bustillos‐Cristales, & Caballero‐Mellado, 2001). In our study, *Burkholderia* and *Ralstonia* showed a positive interaction, while a negative interaction was suspected between *Burkholderia* and *Rickettsia* (Figure 3). The results suggested that *Burkholderia* and *Ralstonia* might be the vital components of the gut bacteria in the three spider species, and the relative abundance of these two bacteria in the spider guts may affect the distribution and abundance of the other bacteria. In regard to other bacteria identified in this study (more than 0.1% of total reads), *Propionibacterium*, *Morganella*, *Providencia*, *Sphingomonas, Chryseobacterium,* and *Corynebacterium* were also found in the guts of mosquitoes (Charan et al., 2016; Manguin, 2013; Muturi et al., 2017). *Lactococcus*, *Lactobacillus*, *Pseudomonas*, *Paracoccus*, *Microbacterium*, *Serratia*, *Achromobacter*, *Bacillus,* and *Agrobacterium* were also found in the guts of Lepidoptera (Chen et al., 2016; Snyman et al., 2016), and *Serratia* are known to contain hemolytic enzymes and could play a potential role in blood digestion (Gaio et al., 2011) and the transmission of other pathogens (Azambuja, Feder, & Garcia, 2004; Gonzalez‐Ceron, Santillan, Rodriguez, Mendez, & Hernandez‐Avila, 2003; Oliver, Russell, Moran, & Hunter, 2003). A report suggested that *Lactobacillus* could be used in prophylactic or therapeutic treatment against natural pathogens (Evans & Lopez, 2004). *Lysobacter*, *Brevibacterium,* and *Proteus* were also identified in the gut of flies (Gupta et al., 2014; Vivero et al., 2016). The bacterium *Acinetobacter* detected in this study was also found in plants that served as food sources for insects (Shi, Lou, & Li, 2010). In conclusion, our results indicated that at the genus level, some of the gut bacteria taxa in three spider species were the same as the gut bacteria of insects.

*Wolbachia* and *Rickettsiella* are endosymbionts existing in spider hosts (Duron, Bouchon, et al., 2008; Goodacre et al., 2006), and the relative abundance of these endosymbionts varies between spider hosts (Zhang et al., 2018). In this study, the relative abundance of *Wolbachia* and *Rickettsiella* in *N*. *albofasciata* (spinning spider) was significantly higher than that in *P*. *laura* (without web) and *P*. *astrigera* (without web) Endosymbionts are widely distributed in the organs and tissues of their arthropod hosts (Pietri, DeBruhl, & Sullivan, 2016; Sicard, Dittmer, Grève, Bouchon, & Braquart‐Varnier, 2014), and whether the high abundance distribution of endosymbionts in the intestinal tissues correlates with the hosts’ digestive function and immune defense is interesting. Furthermore, pathogens often use the host's gut epithelium as an entry site for systematic infections (Engel & Moran, 2013). *Wolbachia* and *Rickettsiella* had a high relative abundance in the digestive tract of *N*. *albofasciata*, and this result suggested that gut epithelium might provide an entry site for infections with these two endosymbionts. Moreover, the results also implied that the gut tissues (epithelial, muscle, and connective tissues) of *N*. *albofasciata* might be one of the best habitats for these two endosymbionts. Therefore, the comparison of the gut histological structures of the three spider species used in this study would provide insights into the relationships between the endosymbiont distribution and the gut histological structure. In addition to these two endosymbionts, *Thermus*, *Amycolatopsis*, *Lactococcus*, *Acinetobacter*, *Microbacterium,* and *Koribacter* from the gut bacterial communities of the three spider species also showed significant differences among the spider species. This result indicated that the composition of gut bacteria might vary slightly according to the difference between hosts and their habitats.

From the results of the Spearman relation network diagram, we found that *Rickettsia* had a negative correlation with other microbes across the gut bacterial communities of the three spider species. As a pathogen, *Rickettsia* can obtain ATP directly from its host (Andersson et al., 1998). The negative correlation of *Rickettsia* and other gut bacteria in our study suggested that gut bacteria might play an important role in preventing *Rickettsia* infection. Although the interaction of symbionts has been examined (Moran, 2006), little is known about the mechanisms of their interactions until now. The diverse microbes in the spider gut may present important functions to spider vital activities. In this study, we also used the Phylogenetic Investigation of Communities by Reconstruction of Unobserved States (PICRUSt) to predict the function of gut microorganisms. The predicted function of gut microbiota in spiders is mostly involved in carbohydrate, amino acid, and energy metabolisms (Figure 4). The functional prediction of the gut microbiota indirectly showed that the gut microbiota might play a vital role in host nutrition and energy supply. Although some functions could be speculated using PICRUSt, many of the actual functions of the gut microbiota are yet to be discovered.

Intestinal microbiota in humans and domesticated mammals include indigenous biota and autochthonous biota (Berg, 1996; Savage, 1977). Researchers have suggested that the definition of gut microbiota in mammals is also appropriate for insects (Dillon & Dillon, 2004). Many insects obtain microbiota from their surrounding environments, such as their food and the skins of animal hosts, and those that can tolerate environmental conditions and the immune functions of the animal gut gain access to a nutrient‐rich environment and a chance for dispersal via the feces (Douglas, 2011). Therefore, some environmental bacteria that can colonize the host's gut in stable quantities would become a part of the host's gut microbiota. In some aquatic insects, the microbial population of 1‐week‐old adults in some aquatic insects are stabilized and less susceptible to colonization by other microbial species (Luxananil, Atomi, Panyim, & Imanaka, 2001; McEwen & Leff, 2001). In this study, we collected the adults of three spider species (two kinds of wandering spiders and one kind of web‐weaving spider) in the same habitat (the cotton field) and maintained the spiders in the laboratory for at least 1 week before dissecting. Although the bacteria from food and the cuticular layers of the spiders can pass into the digestive tract, we suggested that it would be difficult to destroy the stability of the gut bacterial communities of spiders.

However, in addition to the environmental bacteria colonizing the intestinal tract, some bacteria may be contaminants from the environment. In this study, although we tried to reduce this contamination into the minimum through sterile operation, it is difficult to avoid contamination from the spider's integument during the dissecting process. Our spider samples used in this study were collected in the same habitat, to a certain extent, which would reduce the probability of differences in bacterial abundance due to environmental bacterial contamination.

In this study, the PCA plot showed that the bacterial communities were much more similar within species than between species (Figure 1). But as for *P. laura*, one of three replicates on the PCA plot was an outlier replicates, which indicated that significant variability existed within the same spider species. We pooled six guts into each replicate in this study, and three replicates were used for each spider species. In this case, the bacterial composition of each replicate would vary if the bacteria structure of a single gut sample differed from the other five gut samples in one replicate. To some extent, it is possible for the pooled samples that variability existed within a same host species because of some opportunistic reason (such as pathogens invasion). Future studies, we could try to reduce the variability in the future study through increasing the quantity of biological replicates.

Our study only presented some of the gut bacteria of spiders, and more research on the composition and function of spider gut bacteria is necessary.

## CONFLICT OF INTEREST

None declared.

## AUTHOR CONTRIBUTIONS

Guowen Hu and Lihua Zhang contributed equally to this work. Yueli Yun and Yu Peng designed the experiments. Guowen Hu conducted the fieldwork, and Lihua Zhang performed the data analysis. Yueli Yun, Guowen Hu, and Lihua Zhang wrote the manuscript.

## Data Availability

The original data of the gut microbiota relative abundance in spiders are available from the NCBI Sequence Read Archive (SRA) database (Accession number: SRP149550).
