# Supplementary material for: Taking insight into the gut microbiota of three spider species: No characteristic symbiont was found corresponding to the special feeding style of spiders
Source: Ecol Evol. 2019 Jun 23;9(14):8146–56. doi: 10.1002/ece3.5382 (PMC6662400; doi:10.1002/ece3.5382)
Supplement: Supplementary file 1 [file ECE3-9-8146-s001.doc]

**
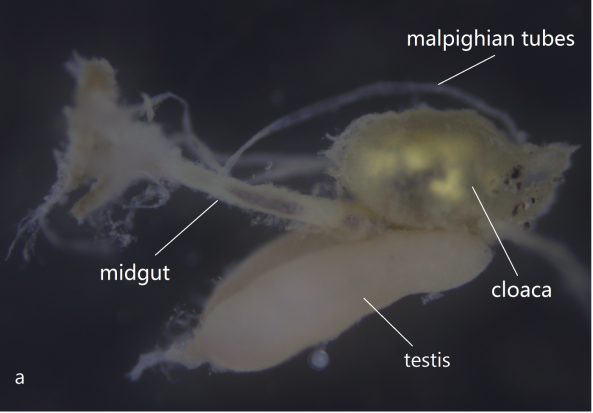

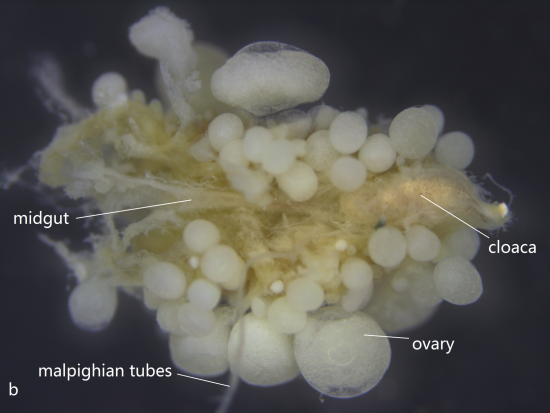
**

**Figure S1** Anatomical drawing of guts in spiders. Figure a was the gut and testis in *N*. *albofasciata*, figure b was the gut and ovary in *P*.*astrigera*.
